# Effects of anesthetic agents on contractions of the pregnant rat myometrium in vivo and in vitro

**DOI:** 10.1007/s00540-020-02866-9

**Published:** 2020-10-24

**Authors:** Motonobu Kimizuka, Yasuyuki Tokinaga, Ryu Azumaguchi, Kosuke Hamada, Satoshi Kazuma, Michiaki Yamakage

**Affiliations:** Department of Anesthesiology, Sapporo Medical University School of Medicine, 291, South 1, West 16, Chuo-ku, Sapporo, Hokkaido 060-8543 Japan

**Keywords:** Myometrial contraction, Dexmedetomidine, Pregnant rat

## Abstract

**Background:**

Several anesthetic agents are used in cesarean sections for both regional and general anesthesia purposes. However, there are no data comparing the in vivo effects of propofol, sevoflurane, and dexmedetomidine on the contraction of the myometrium in pregnant rats. The aim of this study was to investigate the effect of these anesthetic agents on myometrial contraction and elucidate the underlying mechanisms.

**Methods:**

Contraction force and frequency changes in response to propofol, dexmedetomidine, or sevoflurane were evaluated in vivo and in vitro. To test the effect of arachidonic acid on myometrial contraction enhanced by dexmedetomidine, changes in myometrial contraction with dexmedetomidine after administration of indomethacin were evaluated. The amount of phosphorylated myosin phosphatase target subunit 1 (MYPT1) in the membrane fraction was expressed as a percentage of the total fraction by Western blot analysis.

**Results:**

This study demonstrated that dexmedetomidine enhances oxytocin-induced contraction in the myometrium of pregnant rats, whereas propofol and sevoflurane attenuate these contractions. The dexmedetomidine-induced enhancement of myometrial contraction force was abolished by the administration of indomethacin. Propofol did not affect oxytocin-induced MYPT1 phosphorylation, whereas sevoflurane attenuated oxytocin-induced MYPT1 phosphorylation.

**Conclusions:**

Inhibition of myofilament calcium sensitivity may underlie the inhibition of myometrial contraction induced by sevoflurane. Arachidonic acid may play an important role in the enhancement of myometrial contraction induced by dexmedetomidine by increasing myofilament calcium sensitivity. Dexmedetomidine may be used as a sedative agent to promote uterine muscle contraction and suppress bleeding after fetal delivery.

## Introduction

Several anesthetic agents are used in cesarean sections for both regional and general anesthesia purposes. Some anesthetic agents have an inhibitory effect on myometrial contraction and may cause atonic bleeding [1]. A close correlation between myometrial contraction and postpartum hemorrhage during delivery has been reported [2]. Postpartum hemorrhage is the leading cause of maternal death worldwide, responsible for an estimated 100,000 deaths each year [3].

Previous studies have reported that volatile anesthetics such as sevoflurane exert inhibitory effects on myometrial contraction in rats [4–8] and humans [9–12]. Similarly, propofol reportedly inhibits myometrial contraction in rats [13, 14] and humans [15–17]. Tsujiguchi et al*.* reported that the inhibition of contraction by propofol involves inhibition of increases in the intracellular concentration of free Ca^2+^. In other smooth muscles, for example, vascular smooth muscle tone is regulated by changes in both the intracellular Ca^2+^ concentration and myofilament Ca^2+^ sensitivity [18]. Kudo et al*.* reported that contractions in airway smooth muscle are regulated by intracellular Ca^2+^ influx from the sarcoplasmic reticulum or from the extracellular environment via voltage-dependent Ca^2+^ channels (VDCCs) and regulation of calcium sensitivity by the RhoA/Rho kinase pathway [19]. In myometrial smooth muscle, muscle tone is reportedly regulated by changes in intracellular Ca^2+^ concentration through VDCCs [7], but the involvement of myofilament Ca^2+^ sensitivity is unclear.

A possible mechanism to explain the change in myofilament Ca^2+^ sensitivity involves the inactivation of myosin light-chain phosphatase (MLCP) in a process regulated by protein kinase C, arachidonic acid, and Rho-kinase. Myosin phosphatase target subunit 1 (MYPT1) is a targeting subunit of MLCP, and volatile anesthetic agents reportedly inhibit the phosphorylation of MYPT1 [20]. The mechanisms of inhibition are known to differ within the same class of inhalational anesthetic. That is, although both sevoflurane and isoflurane reduce vascular contractions, the underlying mechanisms differ. Isoflurane regulates contractions by altering the intracellular Ca^2+^ concentration, whereas sevoflurane regulates contractions by modulating myofilament Ca^2+^ sensitivity [21]. Therefore, we studied the effects of various anesthetic agents on myofilament calcium sensitivity and myometrial contraction.

Dexmedetomidine is an α_2_-adrenergic agonist that causes minor respiratory depression. α_2_-Adrenergic agonists cause myometrial contraction by inducing an influx of extracellular Ca^2+^, probably through VDCCs and the release of arachidonic acid [22]. Several recent studies reported that dexmedetomidine is effective for treating postpartum shivering in cesarean sections [23–25] and can improve early breastfeeding outcomes [26]. α_2_-Adrenoceptor activation reportedly results in the release of arachidonic acid in vascular smooth muscle [27]. Arachidonic acid in turn inhibits MLCP and increases the Ca^2+^ sensitivity of contractile elements. Therefore, dexmedetomidine may enhance myometrial contraction via its inhibitory effect on MLCP in conjunction with arachidonic acid. However, there are no data comparing the in vivo effects of propofol, sevoflurane, and dexmedetomidine on contraction in the myometrium of pregnant rats. We hypothesized that dexmedetomidine augments myometrial contraction in pregnant rats while propofol and sevoflurane inhibit myometrial contraction. The aim of this study was to investigate the effects of these anesthetic agents on myometrial contraction and elucidate the underlying mechanisms.

## Methods

The effects of dexmedetomidine, propofol, and sevoflurane on myometrial contraction in vitro and in vivo were investigated in pregnant rats.

### Animals

All experiments involving animals were carried out with approval from the Ethics Committee for Animal Research of Sapporo Medical University (permit number: 17–60, 93, 112, 135). Experiments were performed on pregnant Wistar rats, 250–280 g, 19–21 days of gestation. Rats were kept at 24 ± 2 °C under a 12 h/12 h light/dark cycle and maintained on a standard pellet diet with tap water available freely.

### Drugs

All drugs and solutions were prepared immediately before each experiment was performed. Oxytocin (Sigma-Aldrich, St. Louis, MO, USA) was added directly to the organ baths to a final concentration of 20 nM, with reference to a previous study [13]. The effects of propofol (Sigma-Aldrich) were evaluated at cumulative concentrations of 10^−7^ M, 10^−6^ M, and 10^−5^ M. Given that more than 95% of propofol (2–5 × 10^−5^ M) in the blood is bound to plasma protein [28], a free propofol concentration of 4–10 × 10^−7^ M in vitro would be comparable to 9–10 µg/ml of propofol in vivo [29]. Because the therapeutic range of plasma propofol is 2–9 µg/ml, the effective propofol concentrations tested in this study were considered close to the free concentrations clinically observed in serum. The effects of dexmedetomidine (Maruishi, Osaka, Japan) were evaluated at cumulative concentrations of 10^−9^ M, 10^−8^ M, and 10^−7^ M. Ebert et al. reported that the therapeutic plasma concentration of dexmedetomidine for sedation ranges from 0.7 to 1.2 ng/ml [30]. Based on previous studies, we selected dexmedetomidine concentrations that would reach therapeutic plasma concentrations. Sevoflurane (Maruishi) was introduced into the gas mixture using agent-specific vaporizers (Vapor 19.3, Dragerwerk, Lubeck, Germany). All other reagents used in the experiments were of analytical grade.

### Measurement of myometrial contraction

#### In vitro contraction studies

**Fig. 1:**
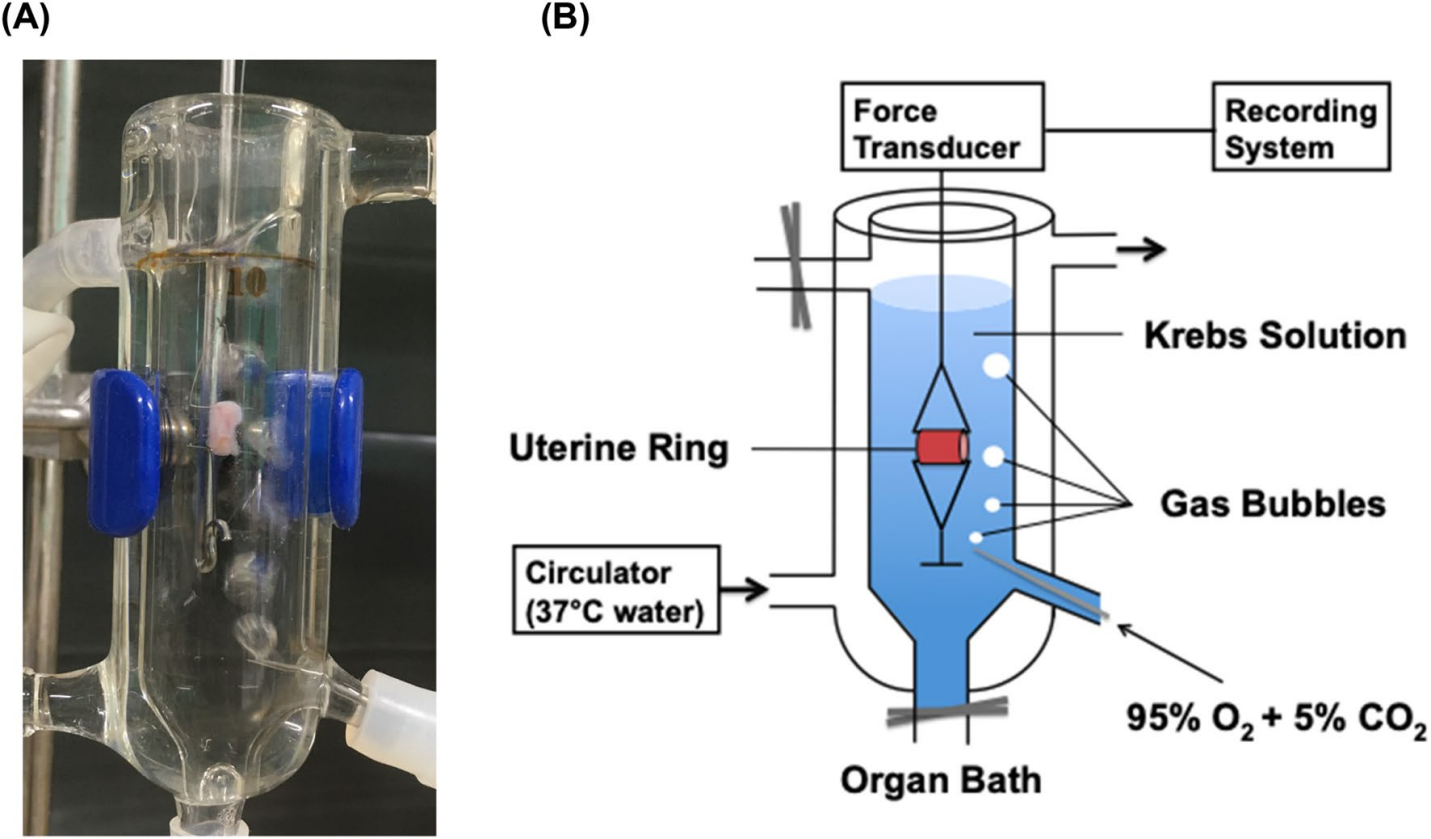
(A) Photo of actual gestational uterine ring, (B) diagram of isolated organ bath system: Isolated organ bath system with an organ bath, heating circulator, force transducer and recording system. Uterine rings (2–3 mm) were sliced from the uterine horns and mounted vertically in an organ bath containing 10 mL Krebs solution bubbled with 95% O_2_ and 5% CO_2_. The temperature of the organ bath was maintained at 37 °C with a heating circulator. The amplitude and frequency of contraction force in the uterine rings were measured using an isometric force transducer (ULA-10GR) and recorded using a recording system (ML846 PowerLab 4/26)

Pregnant Wistar rats were anesthetized with sevoflurane and sacrificed by decapitation. The abdomen of each rat was then opened along the midline; subsequently, the uterine horns were rapidly excised and carefully cleansed of surrounding connective tissues, in reference to previous literature [31]. Embryos and the placenta were gently removed, and 2- to 3-mm-long uterine rings were sliced from the uterine horns and mounted in an organ bath containing 10 mL of Krebs solution (118.2 mM NaCl, 4.6 mM KCl, 2.5 mM CaCl_2_, 1.2 mM MgSO_4_, 1.2 mM KH_2_PO_4_, 24.8 mM NaHCO_3_, 10 mM dextrose), bubbled with 95% O_2_ and 5% CO_2_, and maintained at 37 °C and a pH of approximately 7.40. As shown in Fig. 1, one end of each myometrial strip was connected to a lower hook of the bath, and the other end was connected to a balanced point of a force rod. The resting tension was set at 2.0 g, which was optimal for inducing maximal constriction in our preliminary experiments. Before the start of the experiments, the uterine rings were allowed to equilibrate for 45 min, during which time the Krebs solution was replaced every 15 min.

**Fig. 2:**
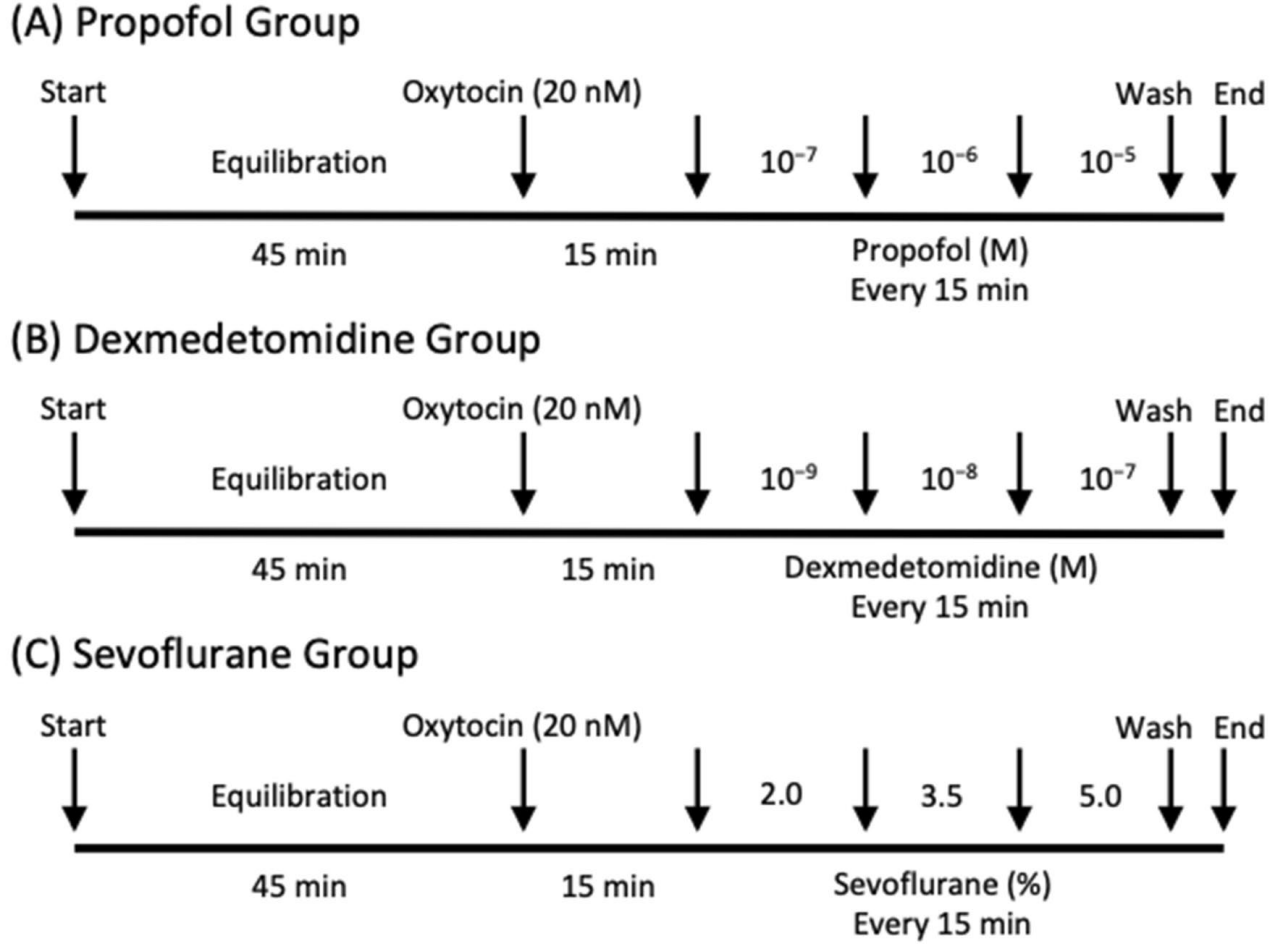
Protocol for in vitro myometrial contraction measurement (n = 10 per group). (A) Protocol for propofol, (B) Protocol for dexmedetomidine, (C) Protocol for sevoflurane. After equilibration, oxytocin (20 nM) was administered to the organ bath. Contraction-force and -frequency changes in response to propofol (10^−7^–10^−5^ M), dexmedetomidine (10^−9^–10^−7^ M), or sevoflurane (2.0, 3.5, 5.0%) were evaluated. In the sevoflurane group, sevoflurane was administered into the gas mix (95% O_2_ and 5% CO_2_). For each concentration of anaesthetic agent, recording was performed for 15 min. Force and frequency of myometrial contractions were measured from the last 10 min of the 15-min exposure

Thirty strips of myometrium were obtained from 15 pregnant rats; each of the two of strips taken per rat was randomly assigned to 1 of the 3 groups (n = 10 per group). The protocol for in vitro myometrial contraction measurement is illustrated in Fig. 2. After equilibration, oxytocin (20 nM) was added to the organ bath; the subsequent response served as a control (100%). After the control contraction occurred, contraction-force and contraction-frequency changes in response to propofol (10^−7^–10^−5^ M), dexmedetomidine (10^−9^–10^−7^ M), or sevoflurane (2.0, 3.5, 5.0%) were evaluated. In the sevoflurane group, sevoflurane was administered via the gas mix (95% O_2_ and 5% CO_2_). For each concentration of the anesthetic agent, measurements were recorded for 15 min.

The amplitude of contraction force in the uterine rings was measured using isometric force transducers (ULA-10GR, MinebeaMitsumi, Nagano, Japan) and recorded using an ML846 PowerLab 4/26 (ADinstruments, Australia). Contraction force was measured by calculating the area under the curve (AUC) of the tension versus time curve. Contraction force and contraction frequency were measured from the last 10 min of the 15-min exposure. The effects of anesthetic agents are expressed as percentages of the control force and frequency of myometrial contraction induced by oxytocin.

Actual values to volatilize volatile anesthetics from organ baths and glass slides may differ from theoretical values. Therefore, the concentrations of the anesthetic agents in the Krebs solution were measured by gas chromatography and determined to be 0.27 ± 0.01 mM and 0.58 ± 0.08 mM at sevoflurane concentrations of 2.4% and 4.8%, respectively (n = 8). It was assumed that the blood concentration of sevoflurane was equal to the Krebs solution dissolved sevoflurane concentration and that the end-expiratory concentration of sevoflurane was equal to the actual dose of sevoflurane, with dialed 2.4% and 4.8% of sevoflurane equivalent to 1.0% and 2.1%, respectively. As sevoflurane volatilized in the organ bath, its concentration decreased by approximately half of the original minimum alveolar concentration (MAC). In a study by Yu et al., sevoflurane was delivered into the gas mixture via calibrated agent-specific vaporizers to aerate the Krebs solution. They concluded that their results would provide a valuable reference for clinical anesthesia research [32]. In our study, we used an organ bath system that supplied anesthetic agents in an identical manner. Thus, as the concentrations were clinically relevant, the findings are considered meaningful.

#### In vivo contraction studies

Twenty-four pregnant Wistar rats were anesthetized with sevoflurane and subjected to tracheotomy. The tail vein was cannulated for intravenous drug administration, and the femoral artery was cannulated for measuring blood pressure.

**Fig. 3:**
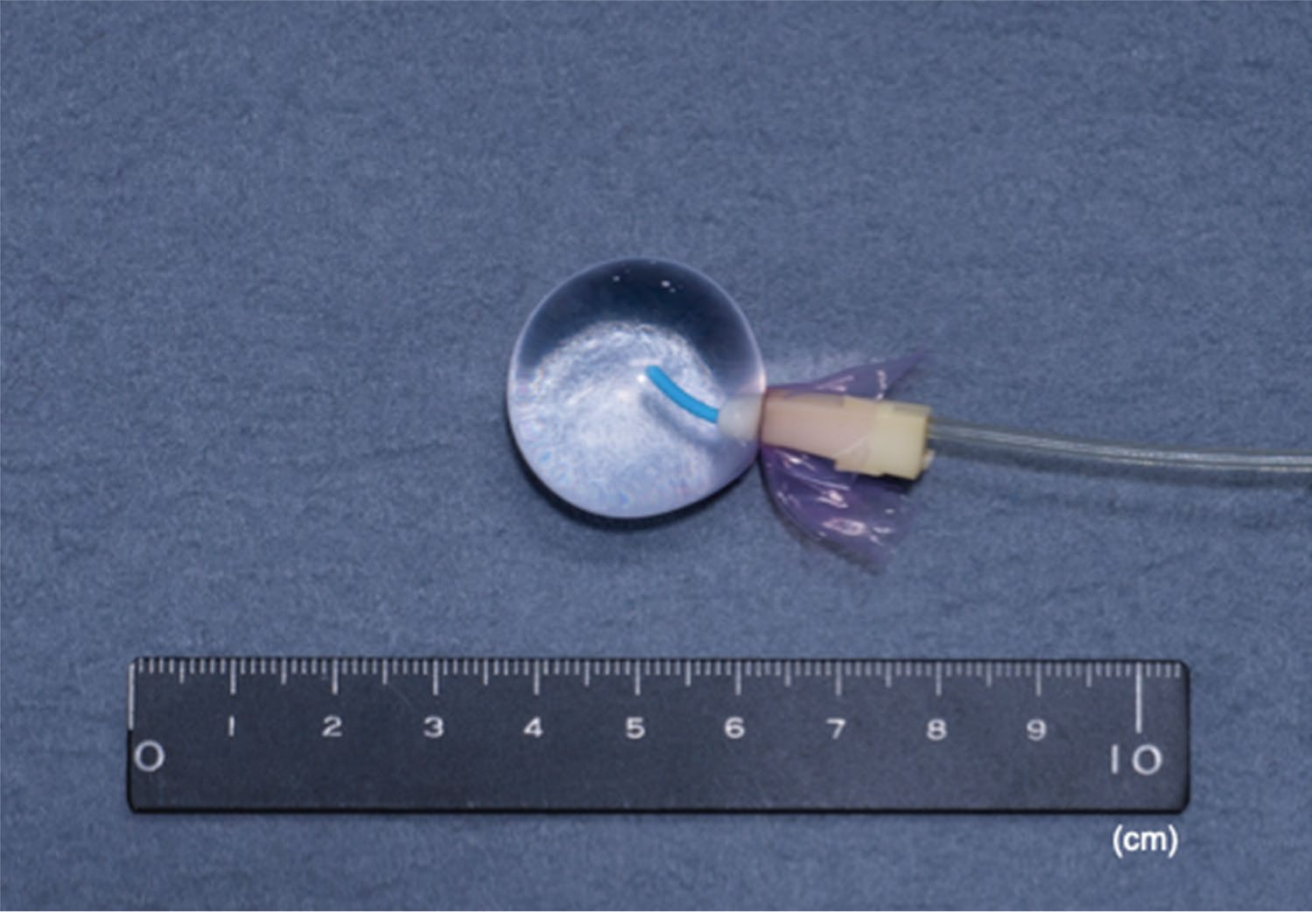
Balloon developed to measure myometrial contractions using a catheter and rubber. The tip of the catheter was covered with rubber, and saline was poured inside. The balloon was then connected to the pressure transducer

A balloon was constructed to measure myometrial contraction using a catheter and rubber (Fig. 3). Next, the abdomen of each rat was opened along the midline; subsequently, an embryo was gently removed, and the balloon was inserted into the myometrium. The wound was closed, and the balloon was connected to the pressure transducer.

**Fig. 4:**
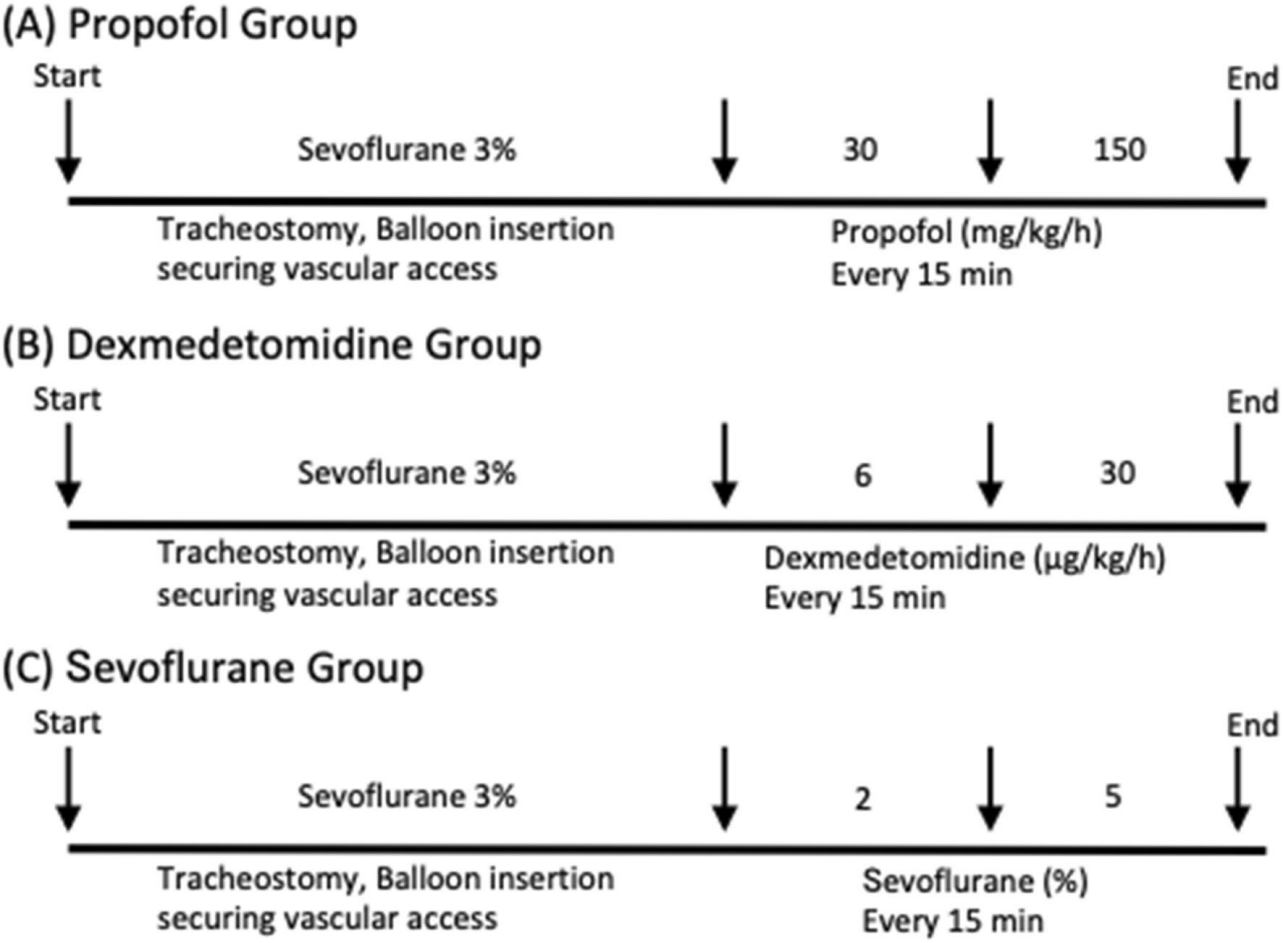
Protocol for in vivo myometrial contraction measurement (n = 8 per group). (A) Protocol for propofol, (B) Protocol for dexmedetomidine, (C) Protocol for sevoflurane. Pregnant rats were anesthetized with sevoflurane subjected to tracheostomy, after which vascular access was obtained and the balloon was inserted in the uterus to measure myometrial contractions. Contraction-force and -frequency changes in response to propofol (30, 150 mg/kg/h), dexmedetomidine (6, 30 μg/kg/h), or sevoflurane (2, 5%) were evaluated. For each concentration of each anesthetic agent, recording was performed for 15 min. Force and frequency of myometrial contractions were measured from the last 10 min of the 15-min exposure

Figure 4 illustrates the protocol for in vivo myometrial contraction measurement*.* After equilibration of myometrial contraction using sevoflurane (3.0%), the anesthetic agent was switched to propofol (30 mg/kg/h), dexmedetomidine (6 µg/kg/h), or sevoflurane (2.0%) (n = 8 per group). Contraction-force and contraction-frequency changes in response to the anesthetic agents were then evaluated. After 15 min of observation, the anesthetic agents were switched to propofol (150 mg/kg/h), dexmedetomidine (30 µg/kg/h), or sevoflurane (5.0%). A neuromuscular blocking agent, rocuronium (Eslax®; MSD Co., Ltd., Tokyo, Japan) was administered to the rats at 5 mg/h (continuous intravenous infusion) after sedation with an anesthetic agent to exclude the effects of skeletal muscles.

### Involvement of arachidonic acid in the effect of dexmedetomidine on myometrial contraction

Indomethacin is a non-steroidal anti-Inflammatory drug that inhibits the arachidonic acid cascade by inhibiting the activity of cyclooxygenase. To examine the effect of arachidonic acid on myometrial contraction enhanced by dexmedetomidine, changes in myometrial contraction with dexmedetomidine after administration of indomethacin were evaluated. Indomethacin (5 mg/kg) was administered by intravenous infusion when the anesthetic agent was changed to dexmedetomidine.

### Western blot analysis

The relationship between the effects of sevoflurane or propofol on uterine muscle contraction and MYPT1 activity was investigated using Western blot analysis. Pregnant Wistar rats were anesthetized with sevoflurane and sacrificed by decapitation. Myometrial tissue strips were collected and treated with oxytocin in the presence or absence of propofol (10^−5^ M), sevoflurane (5.0%), or Y27632 (Rho-kinase inhibitor) (10^−5^ M) for 15 min. After treatment, myometrial tissue strips were homogenized in ice-cold lysis buffer containing HEPES (2-[4-(2-Hydroxyethyl)-1-piperadinyl] ethane sulfonic acid) (50 mM), adjusted to a pH of 7.5, Triton X-100 (1%), NaCl (50 mM), NaF (50 mM), EDTA (5 mM), sodium pyrophosphate (10 mM), phenylmethanesulfonyl fluoride (1 mM), Na_3_VO_4_ (1 mM), leupeptin (100 µg/mL), and aprotinin (10 µg/mL). Homogenates were centrifuged at 1.0 × 10^4^ g for 15 min at 4 °C, and the supernatant was collected. The protein concentration of each supernatant was determined using a Pierce BCA Protein Assay kit (Thermo Fisher Scientific K.K., Yokohama, Japan). Equal amounts of total protein were used for every sample in each experiment. Strips were treated with anti–β-actin antibody (1:1000) as a loading control. Proteins were separated by 7.5% sodium dodecyl sulfate polyacrylamide gel electrophoresis and blotted onto nitrocellulose membranes. The membranes were treated with anti-MYPT1 antibody (1:1000) and anti-phosphorylated MYPT1 antibody (1:1000). The densities of the immunoreactive bands were determined using enhanced chemiluminescence (Amersham Pharmacia Biotech, Piscataway, NJ, USA), and the data were analyzed using an image analysis system (ATTO Corporation, Tokyo, Japan). The amount of phosphorylated MYPT1 in the membrane fraction is expressed as a percentage of the total fraction.

### Statistical analysis

Statistical analysis was performed using GraphPad Prism (version 7.00, GraphPad Software, San Diego, CA, USA). For statistical evaluations, data were analyzed by paired *t*-tests or one-way ANOVA with Tukey’s multiple comparisons test. *P* values < 0.05 were considered significant.

## Results

### In vitro contractility studies

**Fig. 5:**
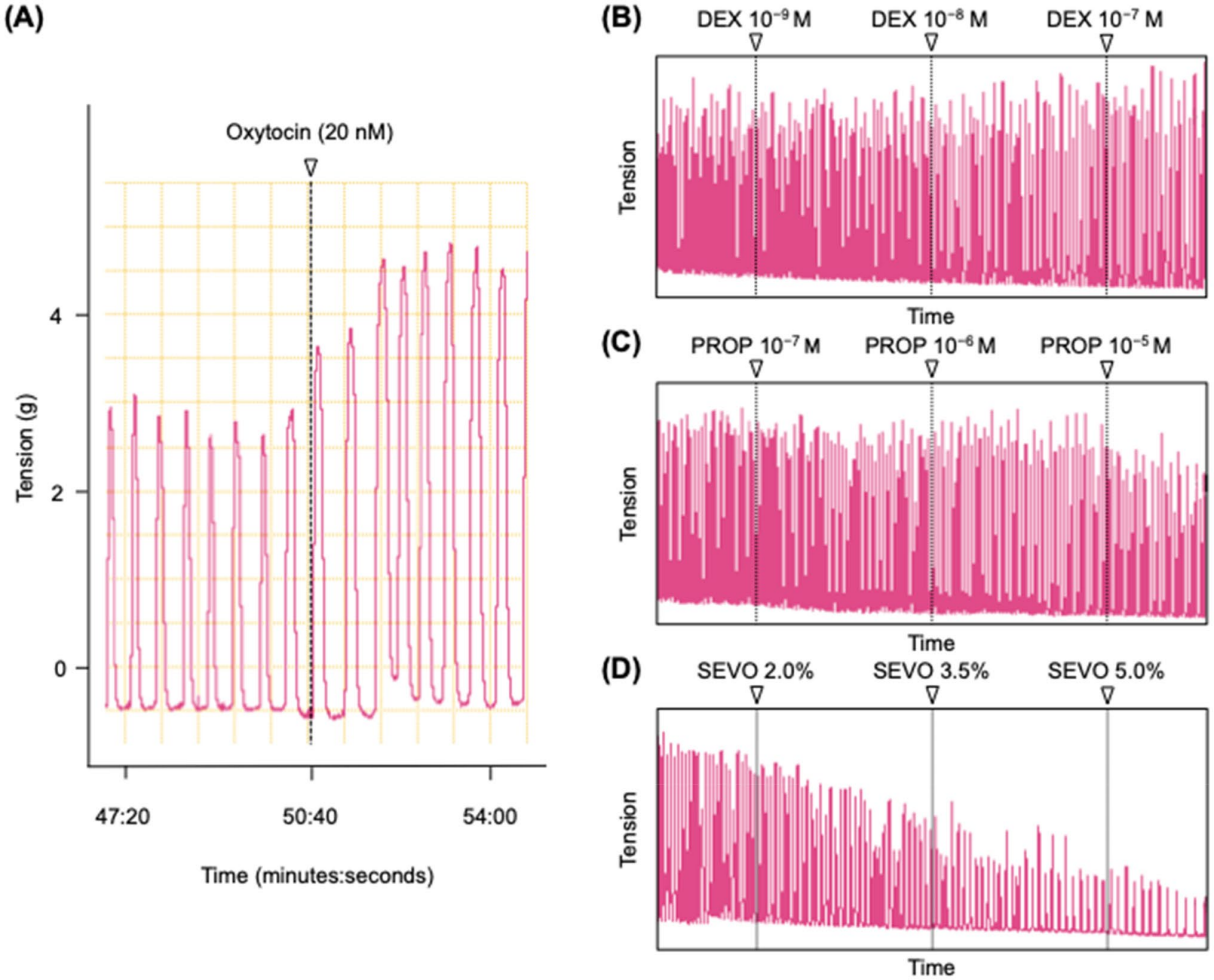
Typical traces of uterine contractions in the organ bath experiments with oxytocin (A) and after the addition of different concentrations of anesthetics (B: dexmedetomidine, C: propofol, D: sevoflurane). DEX: dexmedetomidine, PROP: propofol, SEVO: sevoflurane

**Fig. 6:**
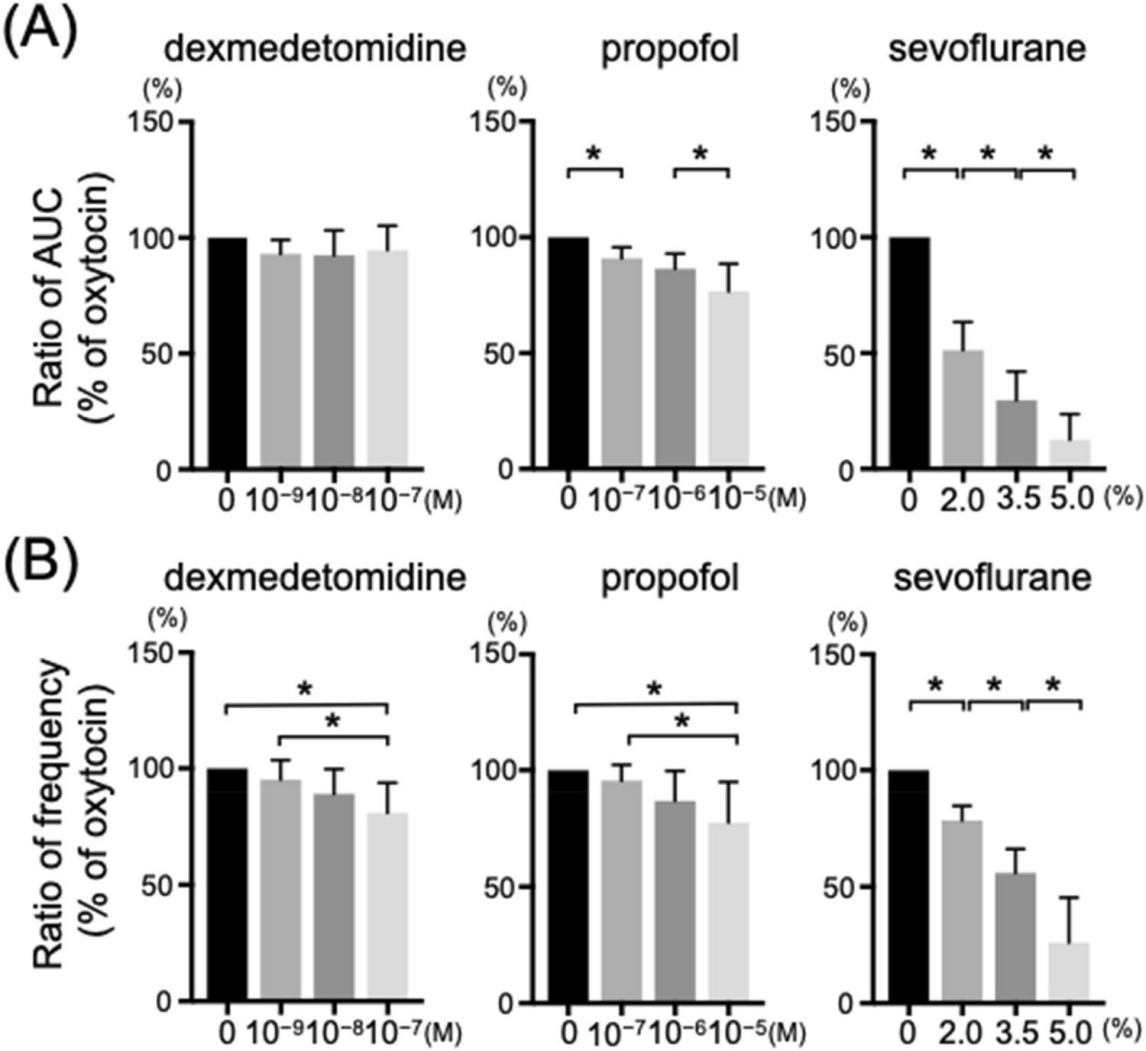
Effects of anesthetic agents on in vitro oxytocin-induced contractions in the myometrium of pregnant rats (n = 10). (A) Ratio of AUC (% of oxytocin [20 nM]); (B) Ratio of frequency (% of oxytocin [20 nM]). AUC: area under the curve, * *P* < 0.05

All uterine strips demonstrated spontaneous contraction in the organ baths. Figure 5 shows typical traces of uterine contractions in the organ bath experiments with oxytocin and after the addition of different concentrations of anesthetics. The frequency of myometrial contraction was significantly decreased by all anesthetic agents. Although the AUCs were significantly decreased by propofol and sevoflurane, no significant difference between any concentration was observed with dexmedetomidine (Fig. 6).

### In vivo contractility studies

**Fig. 7:**
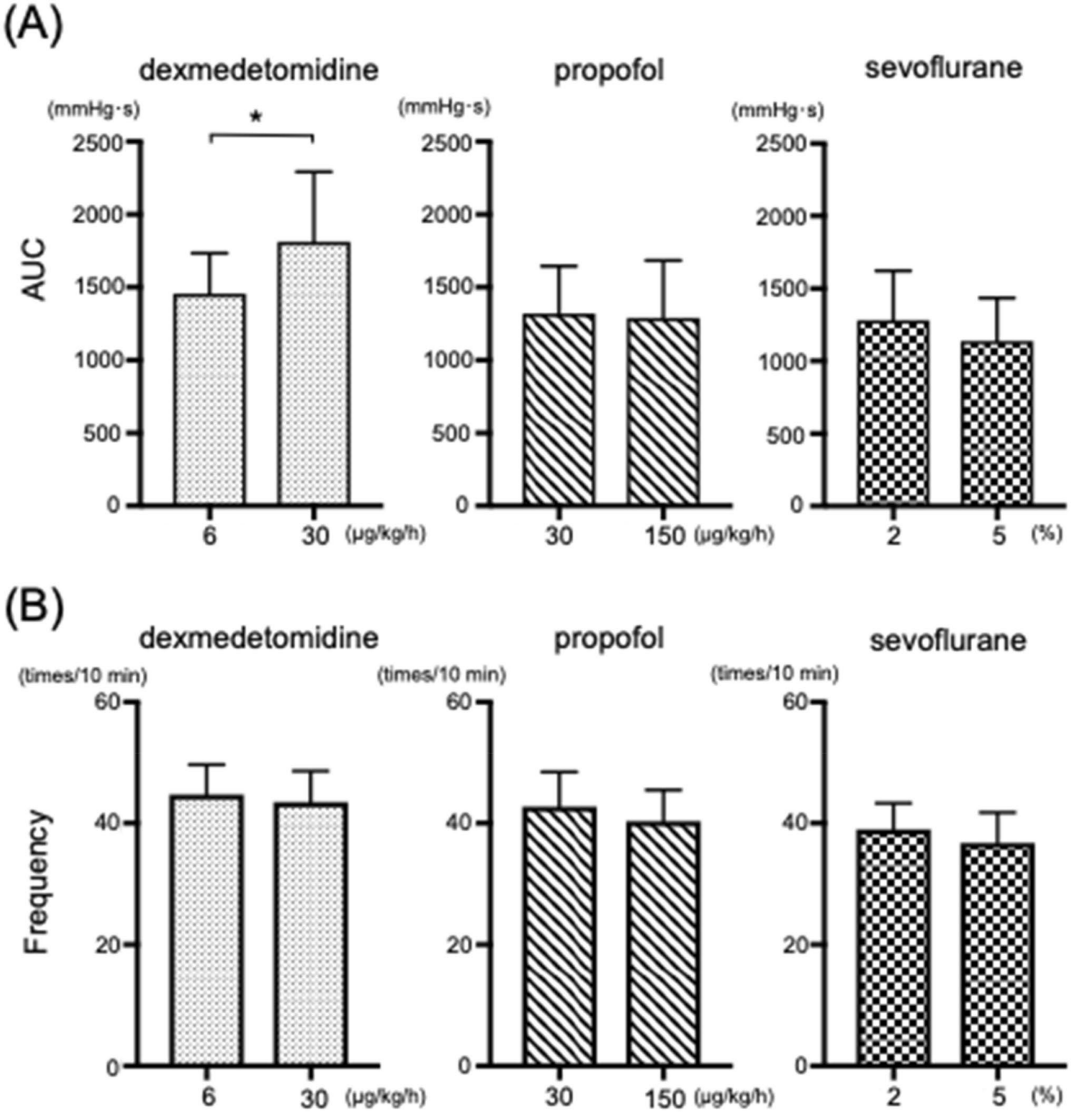
Effects of anesthetics on in vivo oxytocin-induced contractions in the myometrium of pregnant rats (n = 8). (A) AUC: area under the curve; (B) Frequency of contractions. * *P* < 0.05

**Fig. 8:**
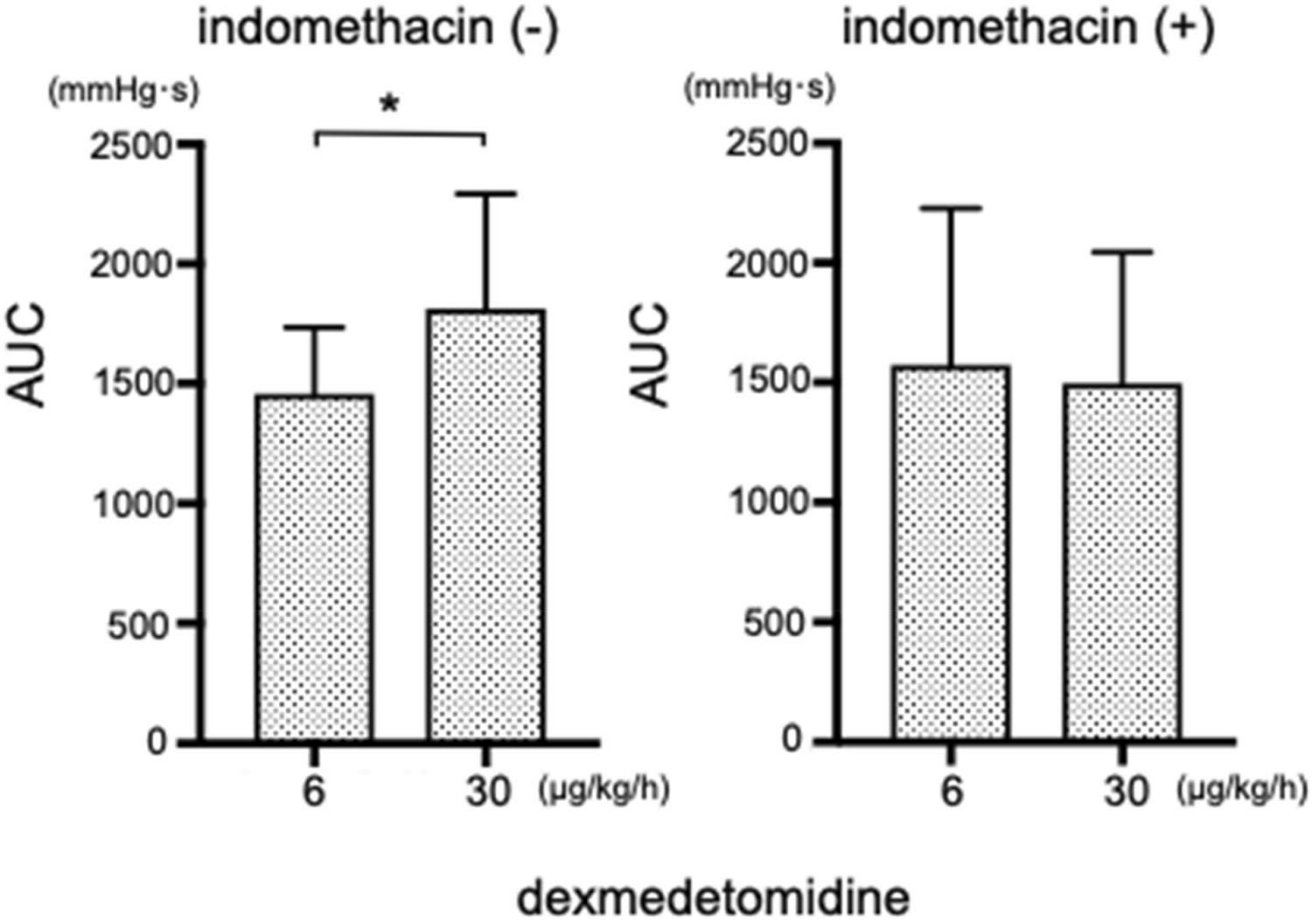
Effect of indomethacin (5 mg/kg) on myometrial contraction enhanced by dexmedetomidine (n = 6). AUC: area under the curve, * *P* < 0.05

There was no difference in the frequency of myometrial contraction induced by any anesthetic agent between the two concentrations examined. The higher concentration of dexmedetomidine significantly enhanced the myometrial contraction force (Fig. 7). The administration of indomethacin abolished the dexmedetomidine-induced enhancement of myometrial contraction force (Fig. 8).

### Western blot analysis

**Fig. 9:**
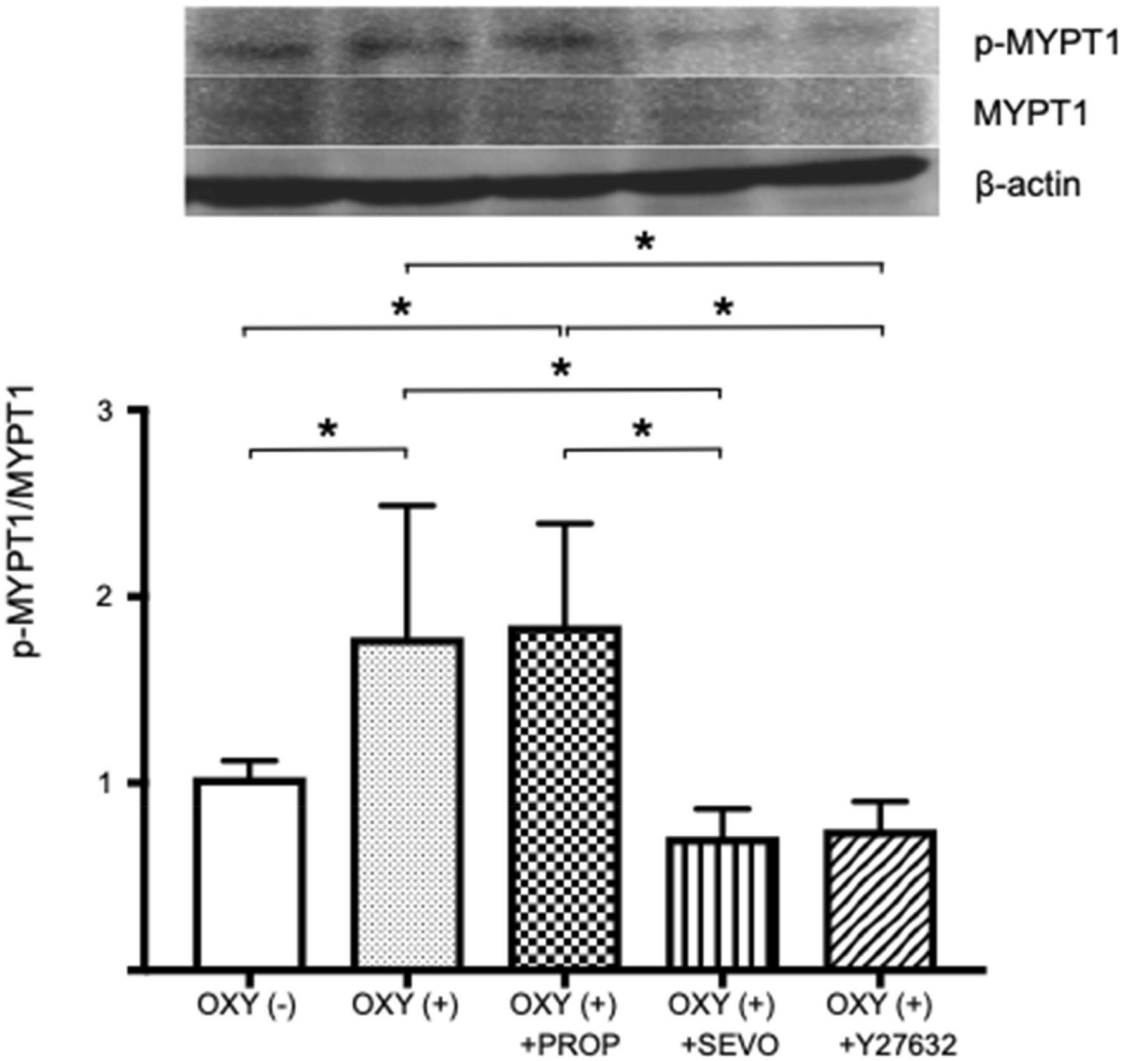
Western blot analysis (n = 6). MYPT1: myosin phosphatase targeting subunit 1, p-MYPT1: phosphorylated MYPT 1, OXY: oxytocin (20 nM), PROP: propofol (10^−5^ M), SEVO: sevoflurane (5.0%), Y27632: Rho-kinase inhibitor (10^−6^ M), * *P* < 0.05

Oxytocin significantly increased the ratio of phosphorylated MYPT1 to total MYPT1 (n = 6 in each group, MD − 0.8, 95% CI − 1.5 to − 0.1, *P* < 0.05). Propofol did not affect oxytocin-induced MYPT1 phosphorylation (n = 6 in each group, MD − 0.1, 95% CI − 0.8 to 1.0, *P* = 0.99), whereas sevoflurane attenuated oxytocin-induced MYPT1 phosphorylation (n = 6 in each group, MD 1.1, 95% CI 0.4 to 1.8, *P* < 0.05) (Fig. 9). β-Actin exhibited no significant differences between groups.

## Discussion

In this study, we demonstrated the effects of dexmedetomidine, propofol, and sevoflurane on myometrial contraction both in vitro and in vivo. We also demonstrated the effect of these anesthetics on the Ca^2+^ sensitivity of the myometrial contraction mechanism.

In our in vitro study, although the contraction force induced by oxytocin was significantly decreased by propofol and sevoflurane, no significant difference was observed with dexmedetomidine. All three anesthetic agents significantly decreased the frequency of myometrial contraction. Previous studies found that propofol reduces oxytocin-induced contraction force and frequency in myometrium isolated from rats [13] and humans [17]. Tsujiguchi et al*.* showed that 10^−5^ M propofol suppressed the 20 nM oxytocin-induced peaks of myometrial contraction by approximately 40%. Interestingly, in the present study, 10^−5^ M propofol reduced the AUC in the 20 nM oxytocin-induced contraction group by 23%. It is difficult to compare these results, however, because the index of myometrial contraction differed, but both results suggest that propofol suppresses myometrial contraction.

Other studies have found that sevoflurane reduces the force and frequency of oxytocin-induced myometrial contraction in myometrium isolated from humans [10] and rats [4, 6]. Dogru et al. reported that the highest measured levels of inhibition of the amplitude and frequency of myometrial contractions with sevoflurane (at 2 MAC) were 84% and 75%, respectively. Although there were methodologic differences between that study and our, such as related to the concentration of oxytocin and the index of contraction, these results were similar to those of the present study.

Previous studies have also found that dexmedetomidine augments spontaneous myometrial contraction in myometrium isolated from humans [33] and rats [31, 34]. Semra et al*.* reported that dexmedetomidine at 10^−9^ M, 10^−7^ M, and 10^−5^ M significantly increased the amplitude, frequency, and AUC of myometrial contraction compared with baseline and control samples. However, they did not use oxytocin to induce myometrial contractions. To the best of our knowledge, no previous studies have examined the effect of dexmedetomidine on oxytocin-induced myometrial contraction in isolated myometrium. In the present in vitro study, dexmedetomidine did not augment oxytocin-induced myometrial contraction. Oxytocin was used in this study at 20 nM so as to potentiate myometrial contraction. It is well known that oxytocin increases spontaneous myometrial contraction [35]. The reason for the difference was that oxytocin had a stronger effect on myometrial contraction than did dexmedetomidine.

In our in vivo study, dexmedetomidine increased the AUC, but no significant differences in AUC induced by propofol or sevoflurane were observed. Many hormones and humoral factors affect the myometrial contraction mechanism similar to oxytocin; thus, there may be mechanisms that involve inhibition of myometrial relaxation. The above reasons explain the differences in the results of the present in vitro and in vivo studies.

Dexmedetomidine, a potent and highly selective α_2_-adrenoceptor agonist, has central and peripheral mechanisms of action. It has been suggested that α_2_-adrenoceptor activation releases arachidonic acid in vascular smooth muscle [23]. Arachidonic acid inhibits MLCP and increases the Ca^2+^ sensitivity of contractile elements [36]. Although the effect of dexmedetomidine on myometrial contraction remains unclear, dexmedetomidine may augment myometrial contraction via the same mechanism. It is well known that indomethacin inhibits cyclooxygenase in the arachidonic acid cascade. In the presence of indomethacin, myometrial contraction induced by oxytocin was inhibited, suggesting that arachidonic acid plays a role in the mechanism by which dexmedetomidine augments myometrial contractions.

In vascular smooth muscle, contraction is regulated by changes in both the intracellular Ca^2+^ concentration and myofilament Ca^2+^ sensitivity. Several studies have reported that contractions in uterine smooth muscle are regulated by changes in intracellular Ca^2+^ concentrations [13, 37]. In contrast, myofilament Ca^2+^ sensitivity has not been studied. Thus, the reason why propofol and sevoflurane did not augment myometrial contractions was investigated from the perspective of myofilament Ca^2+^ sensitivity. In the present study, propofol did not affect oxytocin-induced MYPT1 phosphorylation, whereas sevoflurane attenuated oxytocin-induced MYPT1 phosphorylation. The mechanism of the inhibitory effect of volatile anesthetics on myometrial contraction may differ from that of intravenous anesthetics.

In the present in vivo study, dexmedetomidine enhanced oxytocin-induced myometrial contraction. This suggests that dexmedetomidine enhances myometrial contraction and reduces the amount of bleeding during or after cesarean section. Further studies will be needed to fully characterize the effects of anesthetic agents on myometrial contraction.

There are several limitations in the present study. First, the sedation level of the rats might have differed between anesthetic groups. Although 1 MAC of sevoflurane (2.3%) was used in some previous studies [38], the dose of propofol or dexmedetomidine necessary to obtain a sedation level similar to that of 1 MAC of sevoflurane is unclear. A preliminary experiment was carried out to investigate the minimum concentration needed to sedate rats. The sedative level was evaluated in reference to the method of a previous study [39]. Second, although myofilament Ca^2+^ sensitivity was investigated as the mechanism by which the anesthetic agents regulate myometrial contraction, intracellular Ca^2+^ concentrations were not investigated in this study. It has been reported that myometrial contractions are regulated by changes in intracellular Ca^2+^ concentrations through VDCCs, but the involvement of myofilament Ca^2+^ sensitivity is unclear. Accordingly, the involvement of myofilament Ca^2+^ sensitivity was examined in the present study.

In summary, this study demonstrated that dexmedetomidine enhances oxytocin-induced contraction in the myometrium of pregnant rats, whereas propofol and sevoflurane attenuate contraction. The mechanism underlying the suppression of myometrial contraction differed between propofol and sevoflurane. Inhibition of myofilament Ca^2+^ sensitivity may have inhibited the myometrial contraction induced by sevoflurane. Arachidonic acid may play an important role in the enhancement of myometrial contraction induced by dexmedetomidine via enhancement of myofilament Ca^2+^ sensitivity. Dexmedetomidine could thus be used as a sedative agent after fetal delivery to promote uterine muscle contraction and suppress bleeding.
